# Interactive Effects of Temperature and Grain Moisture Content on Quality Deterioration and Volatile Flavour Evolution in Foxtail Millet During Storage

**DOI:** 10.3390/foods15071157

**Published:** 2026-03-30

**Authors:** Xinyu Hou, Mingjie Sun, Feifan Chen, Fei Han, Yaping Li, Hui Wang, Hong Pan, Quangang Yang, Zhongchen Yang, Yanhong Lou, Yuping Zhuge

**Affiliations:** 1 National Engineering Research Center for Efficient Utilization of Soil and Fertilizer Resources, College of Resources and Environment, Shandong Agricultural University, Daizong Road, Tai’an 271018, China; 2 Shandong Academy of Forestry, Jinan 250014, China

**Keywords:** foxtail millet, storage temperature, grain moisture content, quality, volatile organic compounds

## Abstract

Storage temperature (ST) and grain moisture content (GMC) critically influence cereal quality during storage. However, their interactive effects, the associations among oxidative indicators, quality components and major volatile organic compounds (VOCs) variations in millet during storage are not fully understood. In this study, foxtail millet was stored for 360 days at three STs (−18 °C, 4 °C and 25 °C) and three GMC levels (11.50%, 12.80% and 14.30%). Changes in oxidative indicators (malondialdehyde [MDA], electrical conductivity [EC] and catalase activity [CAT]) and quality components (crude protein [CP], yellow pigment [YP] and soluble sugar [SS]) were monitored. Viscosity characteristics and VOCs were analysed after storage. Under this study, ST was the primary factor driving the changes in oxidative indicators and quality components during the storage stage. The viscosity characteristics of stored millet are primarily influenced by ST, while the changes in major VOCs are mainly affected by ST, GMC, and their interaction effects. Significant negative correlations were observed between EC or MDA and dodecanenitrile and (E/Z)-4-heptenal, whereas the YP, CP, and SS were significantly positively correlated with both compounds. After day 360, the samples stored at −18 °C with 11.5% GMC exhibited 34.05% lower MDA content and 29.55% lower EC than those stored at 25 °C with 14.3% GMC. The treatment better preserved CAT, SS, YP, viscosity characteristics and major VOCs, including (E/Z)-4-heptenal. These findings provide a scientific basis for optimising storage conditions to maintain the nutritional and sensory quality of foxtail millet.

## 1. Introduction

Foxtail millet (*Setaria italica* L.) is increasingly recognised as a climate-resilient and highly nutritious cereal [1]. It contains approximately 61% carbohydrates and 8–14% protein, along with minerals, vitamins and bioactive compounds, making it an essential component of a sustainable diet [2]. The global millet market has shown steady growth in recent years, driven by increasing awareness of nutritional benefits and sustainable agricultural practices [3]. Unlike major staple cereals such as rice and wheat, foxtail millet is typically consumed as porridge or a complementary food with lower daily intake [4]. This lower consumption rate slows inventory turnover and often necessitates long-term storage.

However, prolonged storage inevitably induces ageing and quality deterioration in foxtail millet, triggering oxidative reactions and flavour changes that can compromise its edibility [5]. Lipid oxidation products and secondary aldehydes contribute to the development of rancid or stale off-flavours, which directly affect consumer acceptance and marketability [6]. For instance, grains stored for 270 days at 30 °C exhibited a 241% increase in flavour-related free fatty acid content relative to initial levels, accompanied by severe oxidative damage, indicated by a 124% rise in malondialdehyde content (MDA) [7]. To date, research on foxtail millet storage has predominantly focused on individual changes in either quality components or volatile organic compounds (VOCs) [8]. In contrast, studies addressing the intrinsic mechanisms linking quality and deterioration indicators to the evolution of characteristic flavour compounds in foxtail millet during long-term storage are relatively limited [9]. During storage, the oxidation and degradation of proteins and soluble sugar (SS) are major contributors to the loss of yellow pigment content(YP) and the formation of non-flavour compounds in grains, ultimately affecting their edibility. The flavour of foxtail millet serves as an essential quality indicator during prolonged storage, playing a crucial role in determining sensory acceptability and overall quality perception [10]. Changes in aroma-active compounds during storage result from complex biochemical interactions, in which lipid oxidation, Maillard reaction and enzymatic degradation serve as the primary drivers of the formation, transformation and loss of compounds [11]. These reactions are highly susceptible to environmental factors, including temperature and humidity, which collectively influence the rate and direction of flavour evolution [12]. Advanced analytical techniques, such as gas chromatography–mass spectrometry (GC–MS), headspace solid-phase microextraction (HS-SPME) and electronic nose systems, have facilitated detailed characterisation of volatile profiles in stored grains. Recent studies have shown that the diversity and abundance of volatile compounds exhibit distinct temporal patterns, with specific compounds serving as indicators of different oxidation stages. For instance, hexanal and pentanal are typically associated with early lipid oxidation, while 2,4-decadienal and nonanal are associated with advanced degradation stages [13]. Therefore, changes in protein and lipid stability, driven by environmental factors or inherent grain properties, can significantly influence the quality and flavour of food during storage.

Storage temperature (ST) and grain moisture content (GMC) are widely recognised as the two most critical factors regulating the stability of cereal grains during extended storage [14]. Elevated ST primarily accelerates metabolic activity and chemical reactions. A previous study indicated that after 120 days of storage, tannin content in sorghum grains at 40 °C was approximately 13.9% higher than that in grains stored at 4 °C [15]. Moreover, the free fatty acid content in pearl millet grains stored at 25 °C for 120 days increased by 120.63% compared with initial levels, while grains stored at 5 °C showed an increase of 101.97% [16]. Concurrently, higher GMC considerably promotes microbial growth and activates hydrolytic enzymes, which exacerbate physiological deterioration, activates hydrolytic enzymes such as lipases and amylases, and facilitates non-enzymatic reactions and quality loss during long-term storage [17]. Nevertheless, only a few studies have systematically examined the interactive effects of ST gradient—from freezing through refrigeration to room temperature—and GMC gradients on the dynamic quality changes in millet during long-term storage. Furthermore, although numerous physiological and biochemical indicators have shown changes during storage, it remains unclear which specific indicator serves as the most critical determinant for comprehensively evaluating millet quality.

In this study, the effects of ST and GMC on quality deterioration, viscosity characteristics and VOCs of millet were systematically examined. Specifically, the objectives of this study were as follows: (1) to evaluate the effects of ST and GMC on millet quality during storage, (2) to examine the associations among oxidative indicators, quality componentsand major VOCs, (3) to determine optimal storage conditions within the scope of this study.

## 2. Materials and Methods

### 2.1. Millet Material and Storage Treatments

Foxtail millet cultivar ‘Jigu 19’, developed by the Shandong Academy of Agricultural Sciences, was used in this study. Grain samples were dehulled using a millet huller (JLGJ 4.5; Taizhou Grain Instrument Factory, Taizhou, Zhejiang, China). Before storage, baseline quality assessments were conducted to confirm the uniformity and consistency of the millet samples (Figure S1; Table S1).

The millet samples were divided into three groups and conditioned to target GMCs of 11.5% ± 0.1%, 12.8% ± 0.1% and 14.3% ± 0.1%, respectively, using the equation described in a previous study [18]. All the samples were then sealed in polyethylene bags and stored under three temperature regimes. One group was stored at −18 °C ± 0.5 °C in a refrigerator (iQ 100; Siemens, Munich, Germany), while the remaining groups were stored at 4 °C ± 0.5 °C and 25 °C ± 0.5 °C in temperature-controlled chambers (GXZ-430; Ningbo Southeast Instrument Company, Ningbo, Zhejiang, China). The experimental design comprised nine treatment groups based on combined moisture–temperature combinations: DL (11.5% GMC, −18 °C), DM (11.5% GMC, 4 °C), DH (11.5% GMC, 25 °C), ML (12.8% GMC, −18 °C), MM (12.8% GMC, 4 °C), MH (12.8% GMC, 25 °C), WL (14.3% GMC, −18 °C), WM (14.3% GMC, 4 °C) and WH (14.3% GMC, 25 °C).

Sampling was conducted at 10, 30, 60, 150 and 360 days to assess oxidative indicators and quality components. Viscosity characteristics and VOCs were analysed at the initial time point (day 0) and after 360 days of storage to evaluate ageing effects. At each sampling point, the samples were randomly categorised into three biological replicates, milled through a 100-mesh sieve, immediately flash-frozen in liquid nitrogen and stored at −80 °C until analysis.

### 2.2. Determination of the Oxidative Indicators in Millet

Electrical conductivity (EC) was measured using a calibrated conductivity metre (DDSJ-308F; Leici, Shanghai, China). Catalase activity (CAT) and MDAwere determined using commercial micro assay kits (A007-1 and A003-1, respectively; Nanjing Jiancheng Bioengineering Institute, Nanjing, Jiangsu, China). For each treatment, the samples were randomly selected, and all the measurements were performed in triplicate.

### 2.3. Determination of the Quality Components in Millet

YPwas determined according to a previously described method [19]. Crude protein content (CP)was determined using a Dumas nitrogen analyser (Rapid N Exceed; Elementar, Frankfurt, Germany), with a nitrogen-to-protein conversion factor of 6.25 [20]. SS content was quantified using the previously described anthrone method [21]. Viscosity characteristics were analysed using a rapid viscosity analyser (TechMaster RVA; Perten Instruments, Hägersten, Sweden). Pasting temperature (PT), peak viscosity (PV), trough viscosity (TV), final viscosity (FV), breakdown value (BD = PV − TV) and setback value (SB = FV − PV) were recorded [22]. To ensure representativeness, triplicate samples were randomly selected from each treatment group for subsequent analyses.

### 2.4. Determination of VOCs in Millet

VOCs were extracted using HS-SPME. Briefly, the millet samples were placed in headspace vials containing saturated NaCl solution, and 3-hexanone (10 μg/mL, 20 μL) was added as the internal standard. The HS-SPME extraction procedure was performed according to previously reported methods [23,24].

The extracted VOCs were analysed using a GC–MS system (8890–7000D; Agilent, Santa Clara, CA, USA) equipped with a DB-5MS capillary column (30 m × 0.25 mm × 0.25 μm). Helium was used as the carrier gas. The GC–MS operating conditions, including the oven temperature programme and mass spectrometric parameters, were set as previously described [24]. The analysis was performed using a targeted selected ion monitoring (SIM) mode based on an in-house volatile database established from multiple species, the published literature, commercially available reference standards, and experimentally determined retention index (RI) information [25]. For each compound, one quantitative ion and two to three qualitative ions were selected. The target ions were monitored in time-segmented windows according to their retention time to enhance sensitivity and specificity. Compound annotation was achieved by matching the retention time and characteristic ion fragments with entries in the established database. A compound was considered tentatively identified when (i) its retention time matched the reference value within an acceptable deviation, and (ii) all the selected qualitative ions were detected in the background-corrected mass spectrum. Quantification was performed by MassHunter (version B.08.00; Agilent, Santa Clara, CA, USA) using the selected quantitative ion, with peak area normalised to the internal standard to improve analytical accuracy.

### 2.5. Identification of Major VOCs in Millet

To examine changes in VOCs in millet at day 0 and day 360 under different storage conditions, significant differential accumulation VOCs (DAVs) were identified using an orthogonal partial least-squares discriminant analysis (OPLS-DA) model, with a variable importance in projection (VIP) score ≥ 1.0 and |log_2_FC| ≥ 1.0 [23,24]. To evaluate the potential sensory impact of these compounds, relative odour activity values (*rOAV*) were calculated according to a previous study [24]. The *rOAV* for each compound was determined by dividing its relative concentration (*C_i_*) by the corresponding odour threshold value in the samples (*T_i_*), as per the following equation:

$$rOAV=\;\frac{C_{i}}{T_{i}}$$

### 2.6. Statistical Analysis

All the statistical analyses were conducted using IBM SPSS Statistics (version 26.0; IBM, Armonk, NY, USA). Data were reported as mean ± standard error. Significant differences among means were assessed using Tukey’s multiple range test. To determine the individual and interactive effects of ST and GMC, a two-way analysis of variance (ANOVA) was performed. Differences were considered significant at a 5% probability level (*p* < 0.05). Volcano plots, correlation analyses and Mantel tests were performed using R (version 4.2.0), with the packages ggplot2 (version 3.3.6), psych (version 1.9.12.31) and ggcor (version 0.9.8.1). Data visualisation was performed using GraphPad Prism (version 10.1.2; GraphPad Software, Boston, MA, USA) and Adobe Illustrator (version 29.5; Adobe, San Jose, CA, USA).

## 3. Results

### 3.1. Effects of ST and GMC on Millet Quality

**Figure 1:**
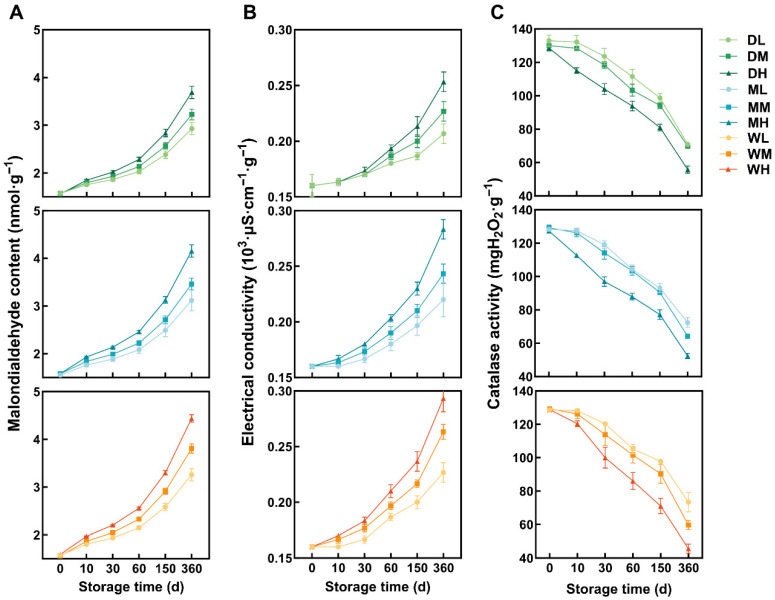
Changes in malondialdehyde content (MDA) (**A**), electrical conductivity (EC) (**B**) and catalase activity (CAT) (**C**) in millet during storage under different treatment conditions.

MDA content increased in all the treatment groups throughout the storage period (Figure 1A). By day 360, MDA content in the DL treatment increased by 86.35% relative to that at day 0, while MDA content in the WH treatment increased by 179.57%, indicating the most severe deterioration. Under identical GMC conditions, ST had a significant effect on MDA accumulation (*p* < 0.05), with higher temperatures accelerating the accumulation. However, at a temperature of −18 °C, GMC did not significantly influence MDA content (*p* > 0.05), whereas at 4 °C, significant moisture-induced variations emerged from day 150 onwards (*p* < 0.05). At 25 °C, 14.3% GMC condition significantly intensified deterioration, resulting in highly significant differences (*p* < 0.01) in MDA among GMC groups from day 10 onwards.

EC increased in all the treatment groups throughout the storage period (Figure 1B). By day 360, the increases relative to day 0 ranged from 29.17% in the DL treatment to 91.30% in the WH treatment, representing the lowest and highest values among all the treatments, respectively. Under identical GMC conditions, the effect of ST on EC (*p* < 0.05) was apparent by day 10 at the highest GMC level (14.3%). This effect was delayed until days 30 and 60 at lower GMC levels (12.8% and 11.5%, respectively), indicating that higher GMC accelerates millet oxidation. However, GMC did not significantly influence EC under different ST conditions throughout the storage period (*p* > 0.05).

CAT declined across all the treatment groups during storage (Figure 1C). By day 360, cumulative reductions relative to day 0 ranged from 43.60% in the ML treatment to 64.60% in the WH treatment, representing the minimum and maximum losses among all the treatments, respectively. At 14.3% GMC, significant temperature-induced variations emerged primarily from day 60 onwards, whereas at 11.5% and 12.8% GMC levels, such variations were significant throughout the storage period. Under all GMC levels, the CAT decline rates at -18 °C and 4 °C were nearly identical, whereas the rate at 25 °C was significantly higher. Two-way ANOVA also demonstrated that elevated ST accelerated the decline in CAT during storage (Table S2).

**Figure 2:**
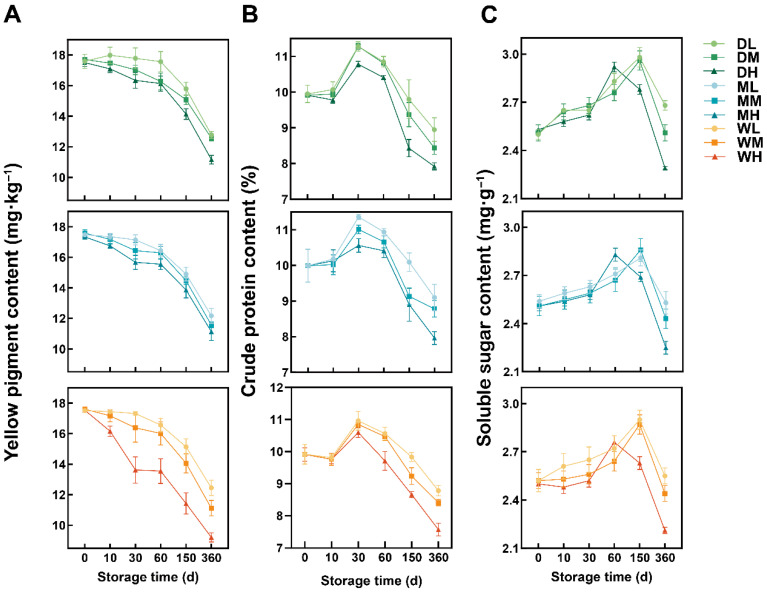
Changes in yellow pigment content (YP)(**A**), crude protein content (CP)(**B**) and soluble sugar content (SS) (**C**) in millet during storage under different treatment conditions.

Under all the treatments, the YP decreased over time (Figure 2A). In the samples stored at 25 °C, the YP in the DH, MH and WH treatments declined by 36.23%, 35.80% and 47.44% by day 360 relative to those at day 0. ST was a critical determining factor in the YP, with significant effects observed by day 10 (*p* < 0.01). Conversely, the impact of GMC became apparent later, primarily from day 30 onwards (*p* < 0.05) (Table S2).

The CP initially increased until day 30, followed by a gradual decline through day 360 (Figure 2B). A two-way ANOVA indicated that ST became a significant factor affecting the CP content starting at day 30 (Table S2). At this time point, CP levels were significantly higher at −18 °C and 4 °C than at 25 °C across all GMC treatments (*p* < 0.05). From day 30 onwards, CP progressively declined across all treatments, with samples stored at 25 °C exhibiting the most rapid degradation. Cumulative reductions of 17.53%, 20.32% and 20.40% were recorded in the DH, MH and WH treatments, respectively.

The SS content in all the treatment groups initially increased and then declined throughout the storage period (Figure 2C). In the samples stored at 25 °C (DH, MH and WH), maximum levels were reached rapidly by day 60, whereas in the samples stored at −18 °C and 4 °C, this peak was delayed until day 150. By day 360, the SS content in the DL treatment was 4.63% higher than that at day 0, whereas the WH treatment showed an 11.8% reduction. Under identical GMC conditions, significant effects of ST (*p* < 0.05) were observed from day 60 onwards, indicating that ST strongly regulated the SS content during storage (Table S2).

### 3.2. Viscosity Characteristics in Foxtail Millet

The viscosity characteristics reflect the quality changes in millet under different STs (Table 1). Two-way ANOVA indicated that ST was the dominant factor influencing millet viscosity characteristics, exerting a significant effect on all the measured parameters (*p* < 0.05). In contrast, GMC and the interaction between ST and GMC exhibited no significant effects (*p* > 0.05) (Table S3). To identify optimal storage conditions, the variations in viscosity characteristics across different ST conditions were further analysed with identical GMC conditions. Comparison of samples stored at different ST groups revealed that an ST of −18 °C effectively preserved the potential viscosity characteristics of millet. Within the same GMC levels, the samples stored at 25 °C (DH, MH and WH) exhibited significantly lower PV and BD than those stored at −18 °C (DL, ML and WL), with reductions of 13.40%, 21.79% and 9.36% for PV and 10.26%, 8.60% and 5.62% for BD, respectively (*p* < 0.05). Furthermore, comparisons with day 0 revealed pronounced quality deterioration in the DH, MH and WH treatments, as evidenced by significant declines in PV (12.46%, 11.27% and 14.10%, respectively) and increased SB. Analysis of the samples at day 360 versus day 0 showed that storage conditions significantly affected PT and FV (*p* < 0.05), while BD remained relatively stable (*p* > 0.05). Notably, the DL and ML treatments exhibited decreases of 11.34% and 11.14% in SB values, respectively, representing significant changes compared with day 0.

### 3.3. Changes in VOCs in Millet Under Storage

**Figure 3:**
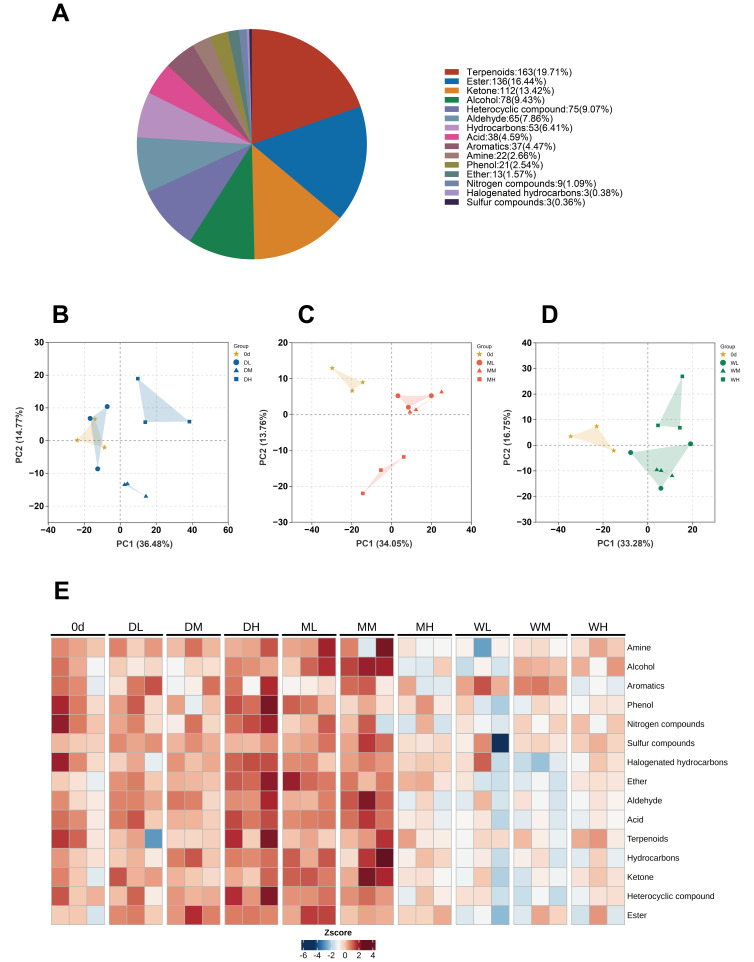
Metabolite analysis of volatile organic compounds (VOCs) in millet during storage: (**A**) Classification and composition of the detected VOCs. (**B**–**D**) Principal component analysis (PCA) of millet samples based on the detected VOCs. (**E**) Heatmap illustrating the relative content distribution of classified VOCs under different treatments at storage days 0 and 360.

To further investigate alterations in VOCs in millet during storage, a comprehensive targeted metabolomics approach based on GC–MS was applied. A total of 828 VOC features were detected and classified into 15 chemical categories, with terpenoids (163 compounds, 19.71%) and esters (136 compounds, 16.44%) being the predominant classes, followed by ketones, alcohols and heterocyclic compounds (Figure 3A).

Principal component analysis (PCA) was performed on all the samples, including quality control samples, based on the quantified volatile metabolite data. PCA score plots were generated for millet samples with GMC levels of 11.5% (PC1 = 36.48%, PC2 = 14.77%; Figure 3B), 12.8% (PC1 = 34.05%, PC2 = 13.76%; Figure 3C) and 14.3% (PC1 = 33.28%, PC2 = 16.75%; Figure 3D) comparing the samples stored for day 0 and day 360 at −18 °C, 4 °C and 25 °C. Each sample replicate clustered closely together, indicating good reproducibility of the experimental method. However, samples from different storage treatments exhibited significant spatial differences in the PCA plots. Except for the DL treatment, the samples collected at day 0 showed greater separation from those obtained after storage, suggesting substantial metabolic differences between pre- and post-storage states under varying GMCs and STs.

Comparisons with the initial samples (day 0) revealed that the VOC profiles underwent significant transformation after storage (Figure 3E). The heatmap clearly illustrates variation in VOCs composition across storage conditions, highlighting distinct moisture-dependent responses and temperature–moisture interactive effects. Notably, GMC emerged as the key factor regulating VOCs abundance in millet after 360 days of storage. Overall, VOCs retention showed a significant downward trend with increasing storage humidity. Aldehydes and acids, which are major secondary products of lipid oxidation and key contributors to rancidity in aged millet, were significantly enriched in the DH, ML and MM treatments. Compared with day 0, terpenoids and esters—essential contributors to characteristic aroma—showed consistent decreases after 360 days of storage, indicating progressive aroma loss during prolonged storage.

### 3.4. Analysis of Major VOCs in Millet During Storage

To enhance discrimination among treatment groups, supervised OPLS-DA was performed. The OPLS-DA score plots showed distinct separations between the samples collected at day 0 and those obtained after 360 days of storage across all the treatments (Figure S2), indicating significant changes in the VOC profiles during storage. Permutation testing (n = 200) confirmed that the models were robust, showing no evidence of overfitting and satisfactory explanatory (R^2^Y) and predictive (Q^2^) capabilities (Figure S2). These results validate the reliability of the OPLS-DA model for subsequent biomarker analysis.

**Figure 4:**
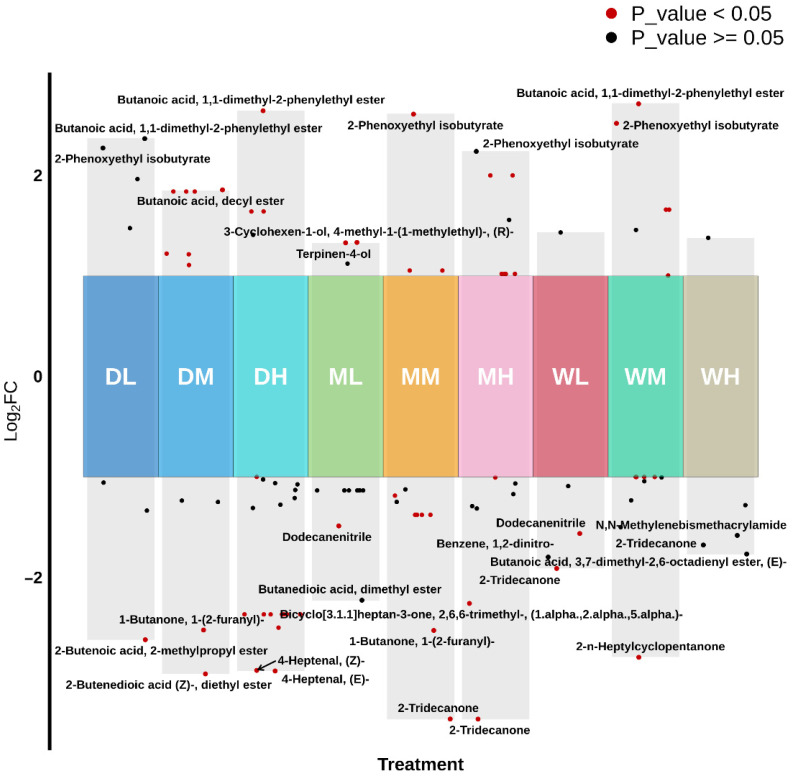
Volcano plots of differential accumulation VOCs (DAVs) in the DL, DM, DH, ML, MM, MH, WL, WM and WH treatments compared with those at day 0.

A total of 61 DAVs were identified based on the following criteria: VIP > 1.0, *p* < 0.05 and |Log_2_FC| > 1.0. Volcano plots depicting the distribution of differential VOCs across multiple treatment groups were generated using log_2_FC values (Figure 4). Distinct differences in DAVs were observed among the millet samples stored for 360 days at −18 °C (L), 4 °C (M) and 25 °C (H) compared with initial samples at day 0. Overall, both the number and magnitude of DAVsincreased with rising ST, with the most pronounced changes observed under high-temperature conditions (25 °C), where more metabolites exhibited significant positive Log_2_ fold changes (*p* < 0.05). 

**Figure 5:**
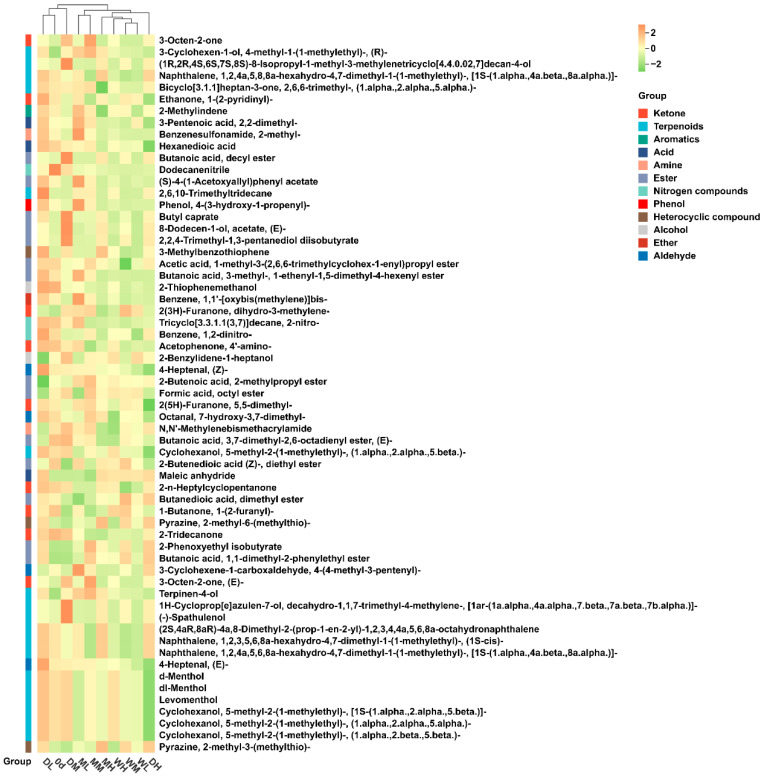
Heatmap of DAVs under different treatments by day 360.

Cluster analysis revealed that the day 0 samples clustered closely with DL and DM treatment samples. Among all the treatments, MH exhibited the highest number of upregulated DAVs, whereas WH and WL showed the fewest. Conversely, DH showed the most extensive downregulation, while DM exhibited the fewest downregulated DAVs (Figure 5).

To identify the key contributors to the VOC profiles, the *rOAVs* of VOCs were calculated. Among the 61 DAVs detected in the millet samples, eight major VOCs exhibited *rOAV* > 1 (Table 2). Of these compounds, Z-4-heptenal contributed the most to the flavour composition of millet, followed by dodecanenitrile and 3-Octen-2-one. Notably, the *rOAV* of dodecanenitrile decreased significantly after storage, whereas the *rOAV* of 3-Octen-2-one, a ketone compound, generally increased during storage. Notably, the most dominant odourant, (Z)-4-heptenal, and the major stale-flavour marker, 3-Octen-2-one, were highly influenced by the interaction of ST and GMC (Table S4).

### 3.5. Multivariate Association and Pattern Analysis

**Figure 6:**
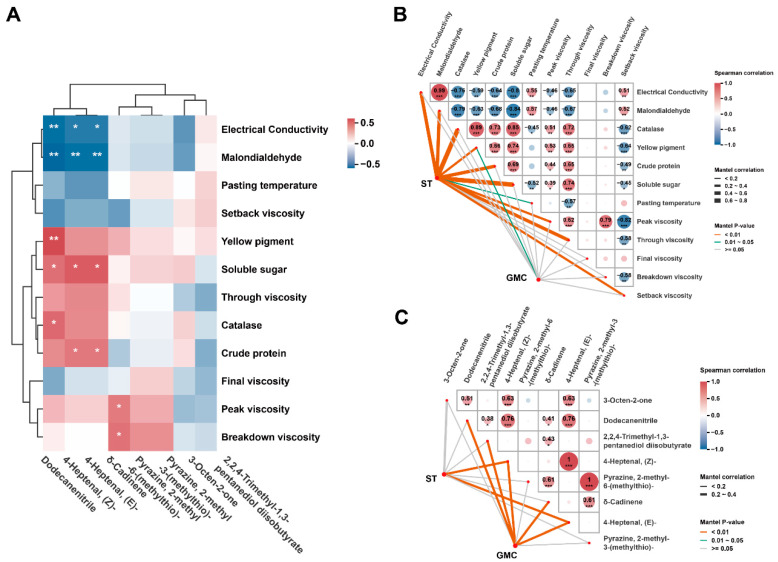
Correlation analysis of quality components and VOCs in millet under different storage conditions: (**A**) Correlations between quality components and VOCs under different storage conditions. Significance levels are indicated as * *p* < 0.05 and ** *p* < 0.01. (**B**) Correlations between quality components of millet under different storage conditions. (**C**) Correlations between VOCs of millet under different storage conditions. Pairwise comparisons between quality components and VOCs are presented, with the colour gradient and circle size denoting Spearman’s correlation coefficients. Significance levels are indicated as * *p* < 0.05, ** *p* < 0.01 and *** *p* < 0.001.

To better understand the relationship between millet quality and storage conditions, the samples at day 360 were analysed for correlations among GMC, ST, oxidative indicators, quality components and major VOCs. Significant negative correlations were observed between specific VOCs (dodecanenitrile, (E/Z)-4-heptenal) and oxidative indicators (EC and MDA) (Figure 6A), indicating an inverse relationship with lipid oxidation severity. Furthermore, quality components—CAT, YP, SS and CP—showed significant positive correlations with dodecanenitrile and (E/Z)-4-heptenal, indicating a positive association between quality retention and these flavour compounds.

Additionally, positive correlations were observed between CAT and YP, CP, SS, PV, TV, while the relationship between EC or MDA and CAT, YP, CP, SS, PV, TV was negative. The positive correlation between EC and MDA was significant. Moreover, YP, CP and SS demonstrated a positive correlation with TV. Furthermore, CAT, SS, EC, MDA, CP, TV, YP, PV and SB demonstrated significant correlations with ST (Mantel *p* < 0.01). Among them, CAT and SS were strongly associated with ST (Mantel’s r 0.63 and 0.65), followed by EC, MDA, CP and TV, with Mantel’s r ranging from 0.4 to 0.6, and YP, PV and SB with Mantel’s r ranging from 0.2 to 0.4 (Figure 6B).

3-Octen-2-one was positively correlated with (E/Z)-4-heptenal, while dodecanenitrile was positively correlated with (E/Z)-4-heptenal, 2,2,4-trimethyl-1,3-pentanediol diisobutyrate, and δ-cadinene. (E/Z)-4-heptenal showed specific correlations with ST (Mantel *p* < 0.01, Mantel’s r ranging from 0.2 to 0.4), while 2,2,4-Trimethyl-1,3-pentanediol diisobutyrate, (E/Z)-4-heptenal and δ-cadinene and dodecanenitrile showed specific correlations with GMC (Mantel *p* < 0.01, Mantel’s r ranging from 0.2 to 0.4) (Figure 6C).

**Figure 7:**
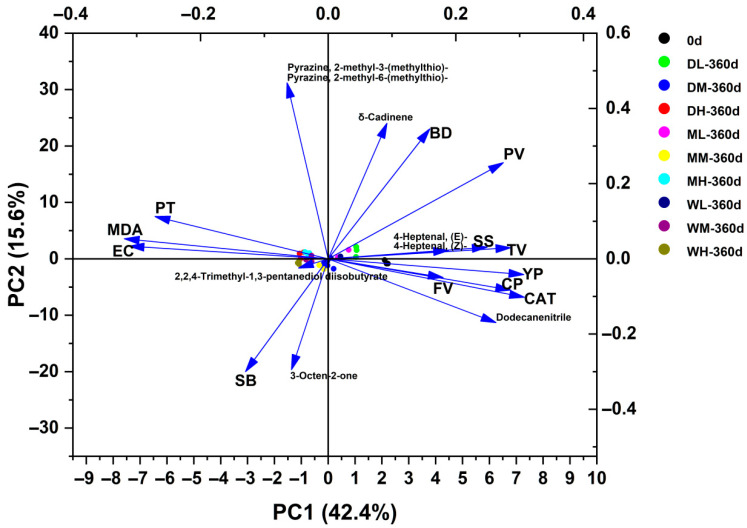
Principal component analysis (PCA) of millet indicators under different treatments.

PCA was conducted to visualise multivariate differences in millet oxidative indicators, quality components and VOC profiles under different ST and GMC conditions. The first two principal components (PC1 and PC2) together accounted for 58.0% of the total variance (Figure 7). Among the samples stored for 360 days, the DL, ML and WL treatments were primarily concentrated on the positive side of both PC1 and PC2, corresponding to higher SS, TV, BD, (E/Z)-4-heptenal and δ-Cadinene. Conversely, the DM, MM, WM, DH, MH and WH treatments were concentrated on the negative side of PC1, clearly separated from the samples from day 0, and were positively correlated with oxidative indicators, viscosity characteristics and major VOCs, including MDA, EC, PT, SB, 2,2,4-trimethyl-1,3-pentanediol diisobutyrate, 3-Octen-2-one and 2-methyl-3/6-(methylthio)-pyrazine.

Overall, the correlation and multivariate analyses provided complementary insights into millet quality changes during storage. The correlation results highlighted significant associations between MDA, EC, CAT, YP, CP, SS, viscosity characteristics and major VOCs, particularly the strong relationship between ST and oxidation-related variables. PCA further demonstrated the overall sample distribution pattern, showing that ST contributed substantially to the primary axis of variation, with −18 °C level clustering distinctly from 4 °C and 25 °C levels.

## 4. Discussion

Foxtail millet is typically stored for extended periods owing to its relatively low daily consumption, making appropriate storage conditions essential for preserving its physicochemical stability and overall edibility. Previous studies have shown that ST and GMC are key determinants of the aging process in cereals such as rice and corn [26,27], suggesting that similar mechanisms may also exist in foxtail millet. Consistent with these observations, this study shows that both ST and GMC significantly influence quality changes in foxtail millet during storage. Among these factors, ST emerged as the dominant driver of quality deterioration, whereas GMC primarily modulated the rate and extent of this process.

Oxidative processes are widely recognised as key contributors in cereals during storage. The onset of deterioration was first evident in oxidative indicators, which showed high temporal sensitivity to storage conditions. Significant differences in MDA and CAT across ST conditions were observed as early as day 10 (Table S1), indicating that lipid peroxidation and enzymatic imbalance were triggered after storage. Elevated ST and GMC likely acted synergistically to accelerate free fatty acid accumulation and subsequent oxidative reactions, resulting in greater MDA accumulation and more pronounced volatile changes under high-ST and high-GMC conditions [9]. Conversely, EC exhibited a delayed response, which was apparent only by day 30 (Figure 1B), consistent with previous findings in rice and wheat. This suggested that membrane structural damage results from cumulative oxidative injury rather than from an immediate response [28]. The rapid decline in CAT observed in the DH, MH and WH treatments (Figure 1C) further compromised antioxidant ability, thereby accelerating MDA accumulation and EC elevation [29]. These findings indicate that oxidative stress is the earliest and primary response to ST, influencing subsequent deterioration in millet quality.

Quality components exhibited delayed responses compared with oxidative indicators. CP and SS increased slightly during early storage and declined thereafter (Figure 2B,C). The transient increase in CP percentage during the early storage stage may partly reflect a relative concentration effect rather than CP synthesis. During storage, respiratory metabolism preferentially consumes carbohydrates and other readily degradable substrates, which can lead to dry matter loss and consequently increase the relative proportion of CP [30,31]. Elevated ST markedly accelerated both the timing and magnitude of YP and SS loss. At 25 °C, heightened metabolic activity promoted Maillard reactions and respiratory consumption, resulting in pronounced declines in CP and SS during later storage stages [32]. YP, primarily carotenoids, declined steadily over time [17], with the most severe losses observed in the WH treatments (Figure 2A). Given that carotenoids contribute to antioxidant defence [33], their rapid depletion under high ST and GMC further exacerbated oxidative deterioration. Conversely, low-temperature storage effectively preserved the loss of YP.

In this study, elevated ST intensified lipid peroxidation, as reflected by the increased MDA and EC, while reduced CAT further compromised antioxidant defence. The quality of millet was jointly regulated by ST and GMC through their influence on metabolic intensity and oxidative degradation. Storage at −18 °C significantly preserved higher SS and YP levels and markedly delayed oxidative damage in comparison with 4 °C and 25 °C. Under these low-temperature conditions, the effect of GMC on quality components was less significant, confirming the dominant role of temperature in maintaining nutritional content during long-term storage.

Elevated temperature can promote partial starch annealing, which reduces granule swelling capacity and consequently lowers PV and BD values. Furthermore, lipid oxidation products and accumulated free fatty acids may interact with amylose to form amylose–lipid complexes. These complexes restrict starch granule swelling and reduce viscosity characteristics [34]. The increase in setback (SB) values observed under higher storage temperatures may be associated with enhanced starch retrogradation, which contributes to firmer texture and reduced palatability [35]. Such structural modifications have been widely reported in stored cereal grains and are consistent with the viscosity trends observed in this study. The observed reductions in PV and BD at 25 °C indicate limited starch swelling, likely due to annealing effects or lipid–amylose complex formation induced by accumulated free fatty acids [36]. Concurrently, the increase in SB values suggests enhanced starch retrogradation, which contributes to a harder texture and reduced palatability [37].

VOC profiles further confirmed sensory deterioration during storage. Storage-induced stress shifted the VOC profiles from freshness-associated aldehydes and esters towards rancidity-related ketones and heterocyclic compounds (Figure 3E). The retention of (Z)-4-heptenal and (E)-4-heptenal in the DL and ML treatments was essential for preserving the characteristic grassy and fatty aroma of fresh millet, whereas the accumulation of 3-octen-2-one at 25 °C reflected hydroperoxide breakdown during linoleic acid oxidation [38]. The significant interaction between ST and GMC on specific volatiles (Table 2) suggests that, while temperature primarily drives lipid oxidation, elevated moisture might promote Maillard reaction pathways that generate roasted or nutty aroma notes, which may contribute to stale sensory characteristics [39,40].

Overall, the deterioration of foxtail millet during storage is strongly associated with ST, which affects its quality through oxidative stress, enzymatic activity reduction and compositional transformations. Within the scope of this study, the DL treatment (11.5% GMC, −18 °C) provided the most favourable preservation effect among the tested conditions. Under this treatment, the MDA content and EC were effectively suppressed; CAT activity was better preserved; and losses of SS, YP and desirable viscosity characteristics were minimised. Moreover, retention of major VOCs, including (E/Z)-4-heptenal, helped preserve sensory quality under this condition. Nevertheless, further studies including multiple cultivars and harvest seasons are required to validate the general applicability of this finding. These findings indicate that coordinated control of ST and GMC can simultaneously mitigate quality deterioration and preserve nutritional and eating quality.

## 5. Conclusions

Under the tested storage temperatures and grain moisture contents, foxtail millet (‘Jigu 19’) underwent progressive biochemical and physicochemical changes over 360 days. During storage, temperature significantly influences the oxidative indicators (MDA, EC and CAT) and quality components (YP, CP and SS), indicating that temperature was primarily related to the onset and progression of deterioration. Low-temperature storage effectively inhibited MDA and EC, better preserved YP, CP, SS, viscosity characteristics and major VOCs of millet over 360 days. Among the tested treatments, the DL treatment (11.5% GMC at −18 °C) showed the most favourable preservation performance within the scope of this experiment. These results provide experimental evidence supporting low-temperature storage strategies for maintaining foxtail millet quality.

## Figures and Tables

**Table 1:** Viscosity characteristics of millet under different storage treatments.

Treatments	Pasting Temperature (°C)	Peak Viscosity (cP)	Trough Viscosity (cP)	Final Viscosity (cP)	Breakdown Value (cP)	Setback Value (cP)
DL	81.69 ± 0.23 b*	2225.67 ± 32.74 a	1264.33 ± 16.41 a	4597.67 ± 18.22 a*	961.33 ± 41.40 a	2372.00 ± 14.98 b*
DM	81.39 ± 0.17 b	1882.67 ± 32.92 b*	1174.67 ± 26.57 a	4505.00 ± 42.48 a*	708.00 ± 59.47 b	2622.33 ± 73.96 a
DH	82.79 ± 0.23 a*	1927.33 ± 21.73 b*	1064.67 ± 22.58 b*	4576.00 ± 45.08 a*	862.67 ± 7.17 ab	2648.67 ± 51.51 a
ML	81.60 ± 0.27 a*	2147.33 ± 63.89 a	1244.33 ± 19.43 a	4524.67 ± 99.16 a*	903.00 ± 69.09 a	2377.33 ± 36.38 b*
MM	82.32 ± 0.22 a*	1888.33 ± 20.48 b*	1105.67 ± 41.29 a*	4613.33 ± 38.11 a*	782.67 ± 32.83 a	2725.00 ± 48.95 a
MH	82.06 ± 0.42 a*	1949.33 ± 16.60 b*	1124.00 ± 31.43 a*	4603.00 ± 25.24 a*	825.33 ± 31.47 a	2653.67 ± 9.17 ab
WL	81.62 ± 0.24 a*	2082.33 ± 42.41 a	1245.67 ± 17.29 a	4612.00 ± 52.84 a*	836.67 ± 29.36 a	2529.67 ± 12.25 b
WM	82.24 ± 0.19 a*	1875.67 ± 39.50 b*	1147.33 ± 26.56 b*	4593.00 ± 39.27 a*	728.33 ± 59.40 a	2717.33 ± 42.23 a
WH	82.12 ± 0.42 a*	1887.33 ± 38.99 b*	1097.67 ± 21.54 b*	4601.00 ± 50.09 a*	789.67 ± 38.83 a	2713.67 ± 68.74 a

Different lowercase letters indicate significant differences (*p* < 0.05) among treatments within the identical GMC under ST conditions. * denotes significant differences between the treatment samples and the day 0 samples (*p* < 0.05).

**Table 2:** Major VOCs of millet under all treatments.

Compounds	Classification	Odour Threshold (μg/kg)	Odour Description	rOAV									
				Day 0	DL	DM	DH	ML	MM	MH	WL	WM	WH
3-Octen-2-one	Ketone	0.00003	Earthy, spicy, herbal, sweet, mushroom, hay, blueberry	861.30 ± 22.61	1139.42 ± 78.52	1825.45 ± 84.81	1277.01 ± 306.74	1036.83 ± 44.91	2118.83 ± 49.17	773.69 ± 65.26	796.31 ± 48.65	800.72 ± 25.70	1195.45 ± 192.92
Dodecanenitrile	Nitrogen compounds	0.00009	Citrus, orange peel, metallic, spicy	1513.58 ± 137.10	768.12 ± 37.41	933.61 ± 231.11	516.54 ± 69.15	594.99 ± 28.21	639.95 ± 35.58	540.51 ± 55.37	484.29 ± 37.22	472.34 ± 25.79	501.51 ± 9.59
2,2,4-Trimethyl-1,3-pentanediol diisobutyrate	Ester	0.014	-	69.31 ± 4.71	89.63 ± 6.05	170.77 ± 8.87	105.20 ± 3.10	65.09 ± 1.35	66.29 ± 1.90	103.49 ± 2.60	48.45 ± 1.60	97.25 ± 3.85	51.98 ± 0.76
(Z)-4-Heptenal	Aldehyde	0.000025	Oily, fatty, green, dairy, milky, creamy	5805.58 ± 118.76	9273.97 ± 475.56	5583.23 ± 233.42	811.20 ± 10.32	5413.66 ± 249.84	5390.63 ± 154.21	4404.04 ± 87.19	2735.51 ± 1030.65	4008.27 ± 113.46	4807.50 ± 91.74
2-Methyl-6-(methylthio)-pyrazine	Heterocyclic compound	0.02	-	0.64 ± 0.35	1.88 ± 0.79	0.30 ± 0.01	2.16 ± 0.28	1.06 ± 0.75	0.80 ± 0.48	2.43 ± 0.05	1.08 ± 0.41	1.97 ± 0.29	0.28 ± 0.01
δ-Cadinene	Terpenoids	0.0015	Thyme, herbal, woody, dry	11.54 ± 0.87	13.52 ± 1.48	8.97 ± 0.76	12.74 ± 0.91	11.16 ± 1.25	5.22 ± 2.18	13.38 ± 1.06	6.31 ± 0.61	5.47 ± 0.66	7.57 ± 1.17
(E)-4-Heptenal	Aldehyde	0.034	Powerful, fresh-fatty-oily odour	4.27 ± 0.09	6.82 ± 0.35	4.11 ± 0.17	0.60 ± 0.01	3.98 ± 0.18	3.96 ± 0.11	3.24 ± 0.06	2.01 ± 0.76	2.95 ± 0.08	3.53 ± 0.07
2-Methyl-3-(methylthio)-pyrazine	Heterocyclic compound	0.001	Roasted meat, nutty, almond, vegetable	12.74 ± 7.01	37.62 ± 15.74	5.97 ± 0.14	43.22 ± 5.64	21.18 ± 14.92	16.06 ± 9.55	48.64 ± 1.01	21.54 ± 8.25	39.33 ± 5.72	5.57 ± 0.18

## Data Availability

The original contributions presented in this study are included in the article/Supplementary Materials. Further inquiries can be directed to the corresponding authors.

## Supplementary Materials

The following supporting information can be downloaded at https://www.mdpi.com/article/10.3390/foods15071157/s1. Figure S1. Baseline oxidative indicators and quality components of millet at day 0. Figure S2. OPLS-DA score plots of differential accumulation of VOCs (DAVs) between each treatment groups at days 360 and 0 and the corresponding permutation test results for each model. Table S1. Viscosity characteristics of millet at day 0. Table S2. Two-way ANOVA of the effects of ST, GMC and their interaction on the oxidative indicators and quality components of millet under different storage periods. Table S3. Two-way ANOVA for the effects of ST, GMC and their interaction on the vis-cosity characteristics of millet. Table S4. Two-way ANOVA for the effects of ST, GMC and their interaction on major VOCs in millet.

## Abbreviations

The following abbreviations are used in this manuscript:

ST	Storage temperature
GMC	Grain moisture content
MDA	Malondialdehyde
EC	Electrical conductivity
CAT	Catalase
YP	Yellow pigment
CP	Crude protein
SS	Soluble sugar
PT	Pasting temperature
PV	Peak viscosity
TV	Trough viscosity
FV	Final viscosity
BD	Breakdown value
SB	Setback value
HS-SPME	Headspace solid-phase microextraction
GC-MS	Gas chromatography–mass spectrometry
VOCs	Volatile organic compounds
DAVs	Differential accumulation VOCs

## References

[1] Mukherjee B., Jha R.K., Sattar A., Dutta S., Bhattacharya U., Samanta S., Huirem B., Singh S.K., Das S., Bal S.K. (2025). Harnessing the potential of millets for climate-resilient and sustainable agriculture. Front. Plant Sci..

[2] Wang L.H., Li Z., Qin J., Huang Y., Zeng X.A., Aadil R.M. (2022). Investigation on the impact of quality characteristics and storage stability of foxtail millet induced by air cold plasma. Front. Nutr..

[3] Muskan F., Redhu M., Redhu S., Rahimi M. (2025). Millets in the global market: A critical review of challenges and opportunities. Food Prod. Process. Nutr..

[4] McDowell R., Banda L., Bean S.R., Morris G.P., Rhodes D.H. (2024). Grain yellowness is an effective predictor of carotenoid content in global sorghum populations. Sci. Rep..

[5] Li D., Xing J., Wang P., Wang L., Zhang Y., He R., Ouyang D., Wan Y., Luo X. (2025). Electron beam irradiation inhibits high-moisture rice quality deterioration during storage. J. Cereal Sci..

[6] Esmi F., Izadpanah M., Soleimaninejad M., Noori A.A., Soltan J. (2025). Off-flavour challenges in plant proteins: Mechanisms, advanced oxidation technologies, and opportunities for flavour improvement—A critical review. Food Bioprocess Technol..

[7] Tian P.P., Lv Y.Y., Yuan W.J., Zhang S.B., Hu Y.S. (2019). Effect of artificial aging on wheat quality deterioration during storage. J. Stored Prod. Res..

[8] Sun Y., Liu S., Wen Q., Wang H., Lu B., Zhu X., Jiang L. (2025). Analysis of the quality and flavor changes of soybean-based meat analogue during storage based on gas chromatography–mass spectrometry and electronic nose. Food Chem. X.

[9] Wu T., Zhou H., Ma Z., Wang S., Wang C., Shen Q., Zhao Q. (2025). Proteomic insights into lipid degradation and volatile compound changes during foxtail millet storage. Food Chem..

[10] Zhu D., Zheng X., Dong H., Liu X., Hu X., Chen M., Liu X., Shao Y. (2025). Effects of storage on volatile organic components and physiological properties of different storage-tolerant rice varieties. Food Chem. X.

[11] Shi Y., Li J., Zhou L., Zhang J., Feng X., Xing W., Tang C., Bai Y. (2025). Exploring the contribution of phosphatidylcholine and triglyceride on the formation of beef aroma-active compounds with thermal oxidation system. Curr. Res. Food Sci..

[12] Nickhil C., Singh R., Deka S.C., Srivastava B. (2025). Structural and physicochemical changes in protein and starch during finger millet storage. Discov. Food.

[13] Grebenteuch S., Kroh L.W., Drusch S., Rohn S. (2021). Formation of secondary and tertiary volatile compounds resulting from the lipid oxidation of rapeseed oil. Foods.

[14] Liu J., Li P. (2021). Control and real-time data acquisition of an experimental platform for stored grain aeration study. Sensors.

[15] de Oliveira K.G., Queiroz V.A.V., de Almeida Carlos L., de Morais Cardoso L., Pinheiro-Sant’Ana H.M., Anunciação P.C., de Menezes C.B., da Silva E.C., Barros F. (2017). Effect of the storage time and temperature on phenolic compounds of sorghum grain and flour. Food Chem..

[16] Selvan S.S., Mohapatra D., Anakkallan S., Kate A., Tripathi M.K., Singh K., Kar A. (2022). Oxidation kinetics and ANN model for shelf life estimation of pearl millet (*Pennisetum glaucum* L.) grains during storage. J. Food Process. Preserv..

[17] Wang R., Liu L., Guo Y., He X., Lu Q. (2020). Effects of deterioration and mildewing on the quality of wheat seeds with different moisture contents during storage. RSC Adv..

[18] Joshi J., Rao P.S. (2024). Predictive modeling of allowable storage time of finger millet grains using artificial neural network and support vector regression approaches. J. Food Eng..

[19] Liu Q., Hu D., Qiao Y., Zai X., Hao X., Zong Y., Zhang D., Shi X., Zhang F., Li P. (2025). Phyllosphere microbes in foxtail millet primarily affect quality by modulating coloration and bitter compounds. Microbiome.

[20] Cain E., Hodgkinson S.M., McNabb W., Brodkorb A., Giblin L., Hayes M. (2025). Protein extraction from Buckwheat, Chondrus crispus, and Spelt and assessment of nutritional benefits and limitations in vitro. npj Sci. Food.

[21] Li W., Zhang Z., Chen R., Sun L., Lai X., Li Q., Chen Z. (2025). Metabolomics-based analysis of the effects of differences in soluble sugars on the sweetness quality of six major tea types in China. Food Funct..

[22] Sun M., Kang X., Wang T., Fan L., Wang H., Pan H., Yang Q., Liu H., Lou Y., Zhuge Y. (2021). Genotypic diversity of quality traits in Chinese foxtail millet (*Setaria italica* L.) and the establishment of a quality evaluation system. Food Chem..

[23] Yan Z., Li Z., Wang H., Dou H., Song J., Ji F., Yang Y., Lin D. (2025). Volatile metabolomics analysis reveals the flavor response of different parts of celery to ultraviolet radiation. Food Chem. X.

[24] Wu Q., Ma W., Liu S., Zhou F., Wu H., Wang X., Yang G., Fang Y. (2025). Urolithin A-producing *Limosilactobacillus fermentum* FUA033 fermentation significantly improves the sensory and antioxidant properties of strawberry juice. Food Chem. X.

[25] Yuan H., Cao G., Hou X., Huang M., Du P., Tan T., Zhang Y., Zhou H., Liu X., Liu L. (2022). Development of a widely targeted volatilomics method for profiling volatilomes in plants. Mol. Plant.

[26] Zhu D., Wang T., Liu X., Bi J., Zhang W., Zeng X., Wang P., Shu Z. (2024). Quality changes in Chinese high-quality indica rice under different storage temperatures with varying initial moisture contents. Front. Nutr..

[27] Mabasso G.A., Resende O., Souza D.G., dos Santos Rosa E., de Almeida A.B., Bessa J.F.V., Célia J.A., Leite J.M., Leite L.F. (2024). Physical properties and quality of corn grains stored at different initial moisture contents under hermetic and non-hermetic conditions. J. Stored Prod. Res..

[28] Padhiar D., Kaur S., Jha U.C., Prasad P.V.V., Sharma K.D., Kumar S., Parida S.K., Siddique K.H.M., Nayyar H. (2025). Differential resilience of chickpea’s reproductive organs to cold stress across developmental stages: Insights into antioxidant strategies for enhanced fertility. Front. Plant Sci..

[29] Strelec I., Mrša V., Simović D.Š., Petrović J., Zahorec J., Budžaki S. (2024). Biochemical and quality parameter changes of wheat grains during one-year storage under different storage conditions. Sustainability.

[30] Russo G.L., Langellotti A.L., Buonocunto G., Puleo S., Di Monaco R., Anastasio A., Vuoso V., Smaldone G., Baselice M., Capuano F. (2023). The sous vide cooking of Mediterranean mussel (*Mytilus galloprovincialis*): Safety and quality assessment. Foods.

[31] Ssali R., Carey E., Imoro S., Low J.W., Dery E.K., Boakye A., Oduro I., Omodamiro R.M., Yusuf H.L., Etwire E. (2021). Fried sweetpotato user preferences identified in Nigeria and Ghana and implications for trait evaluation. Int. J. Food Sci. Technol..

[32] Hsu T.Y., Yang K.M., Chiang Y.C., Lin L.Y., Chiang P.Y. (2024). The browning properties, antioxidant activity, and α-glucosidase inhibitory improvement of aged oranges (*Citrus sinensis*). Foods.

[33] Ke Q.B., Kang L., Kim H.S., Xie T., Liu C., Ji C.Y., Kim S.H., Park W.S., Ahn M.-J., Wang S. (2019). Down-regulation of lycopene ε-cyclase expression in transgenic sweetpotato plants increases the carotenoid content and tolerance to abiotic stress. Plant Sci..

[34] Qu C., Yu D., Jing Z., Gu S., Wang Y., Xie W., Wu Q. (2025). Comparative study on structural characterization, physicochemical properties, and in vitro probiotic activities of resistant starch from different varieties of Euryale ferox. Food Chem. X.

[35] Jiang Y., Chen Y., Zhao C., Liu G., Shi Y., Zhao L., Wang Y., Wang W., Xu K., Li G. (2022). The starch physicochemical properties between superior and inferior grains of japonica rice under panicle nitrogen fertilizer determine the difference in eating quality. Foods.

[36] Mathobo V.M., Onipe O.O., Silungwe H., Ramashia S.E., Anyasi T.A. (2023). Optimisation of the techno-functional and thermal properties of heat moisture treated Bambara groundnut starch using response surface methodology. Sci. Rep..

[37] Sozer N., Dogan H., Kokini J.L. (2011). Textural properties and their correlation to cell structure in porous food materials. J. Agric. Food Chem..

[38] Wang Z., Ma J., Ma G., Yu Q., Han L., Zhang L. (2025). The mitochondrial functional characteristics and microstructure play an important role in yak meat color during wet curing. Food Chem. X.

[39] Zhang K., Gao L., Zhang C., Feng T., Zhuang H. (2022). Analysis of volatile flavor compounds of corn under different treatments by GC-MS and GC-IMS. Front. Chem..

[40] Wu D.T., Li W.X., Wan J.J., Hu Y.C., Gan R.Y., Zou L. (2023). A comprehensive review of pea (*Pisum sativum* L.): Chemical composition, processing, health benefits, and food applications. Foods.

