# Supplementary material for: Interactive Effects of Temperature and Grain Moisture Content on Quality Deterioration and Volatile Flavour Evolution in Foxtail Millet During Storage
Source: Foods. 2026 Mar 30;15(7):1157. doi: 10.3390/foods15071157 (PMC13072984; doi:10.3390/foods15071157)
Supplement: Supplementary file 1 [file foods-15-01157-s001.zip › foods-4169669-supplementary.pdf]

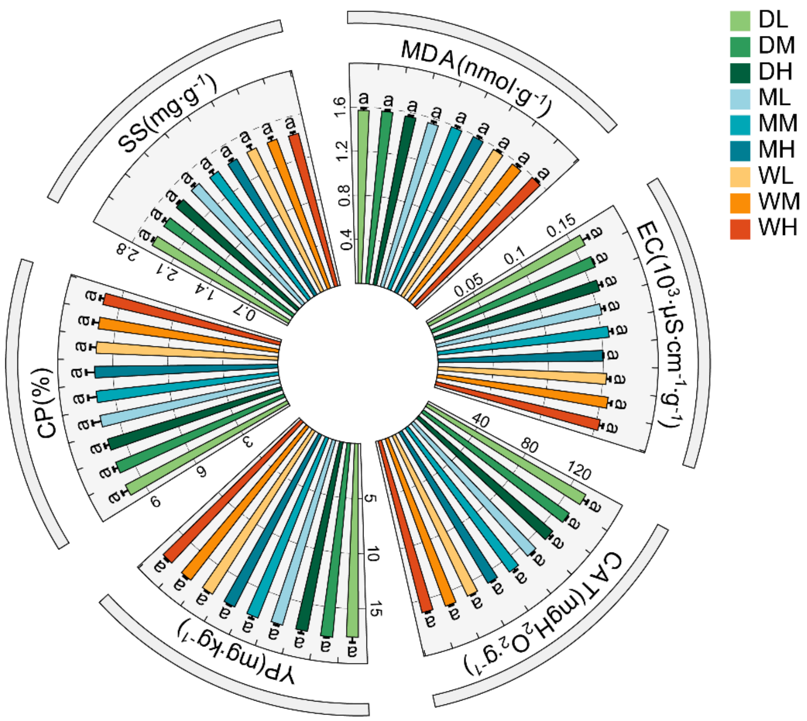

Figure S1. Baseline oxidative indicators and quality components of millet at day 0.

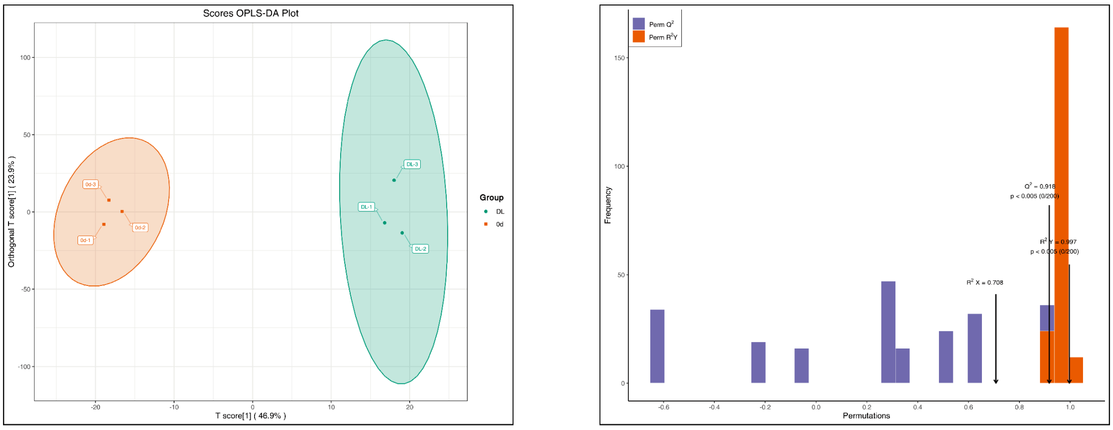

DL_vs_0d

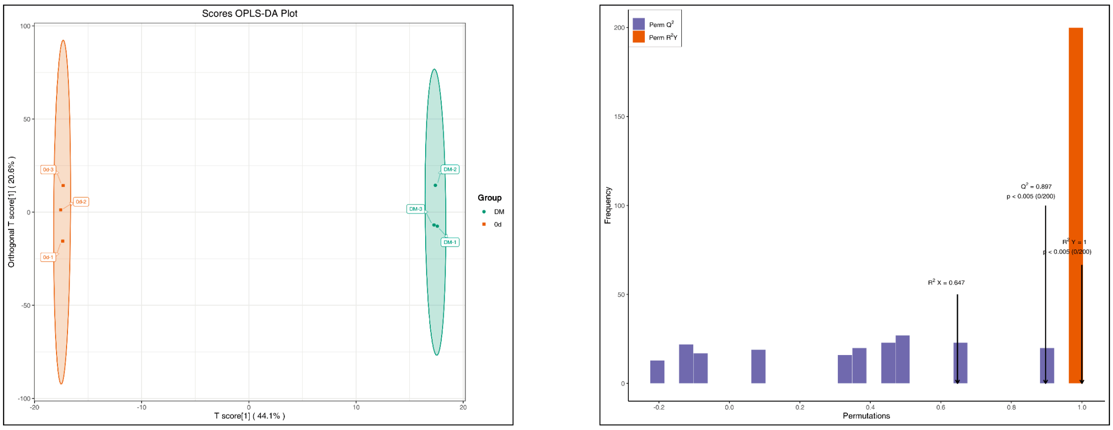

DM_vs_0d

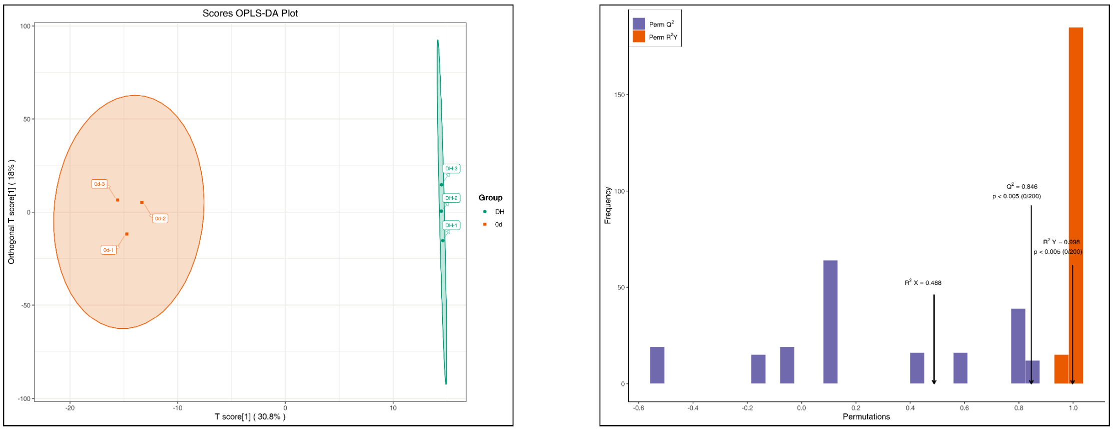

DH_vs_0d

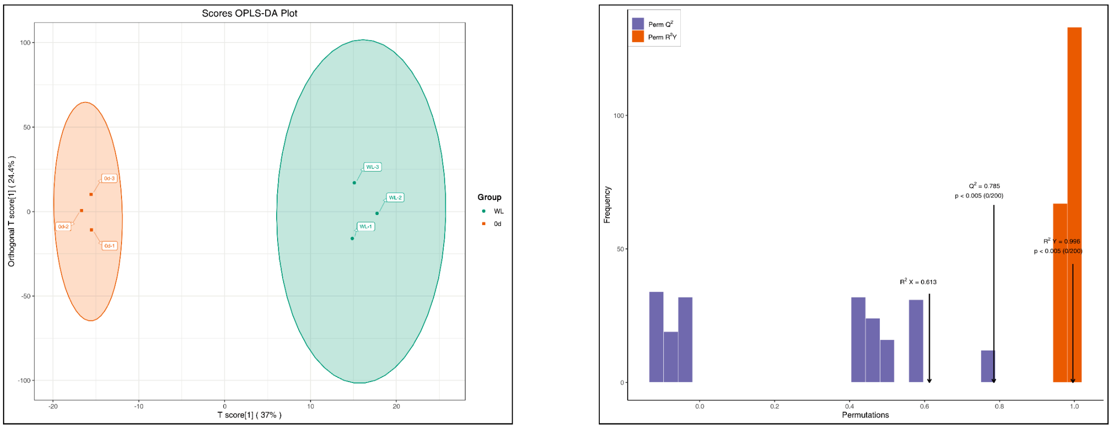

WL_vs_0d

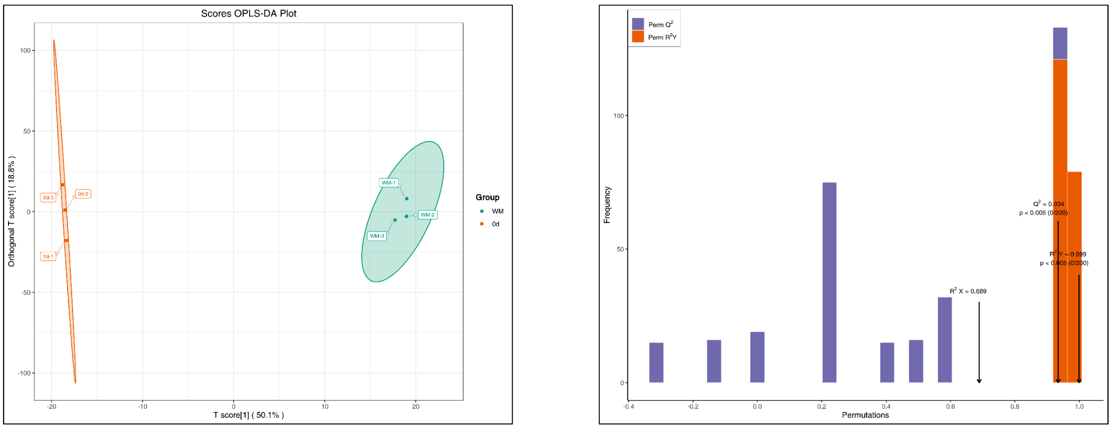

WM_vs_0d

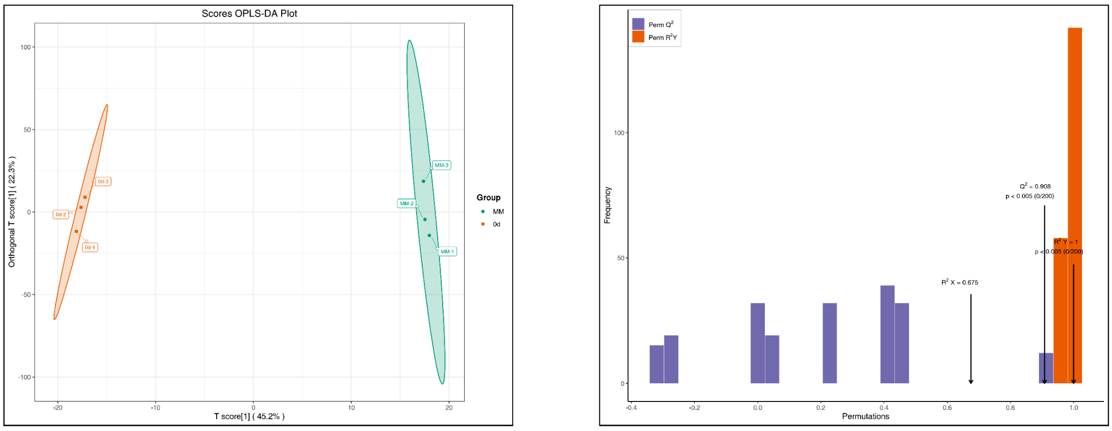

WH_vs_0d

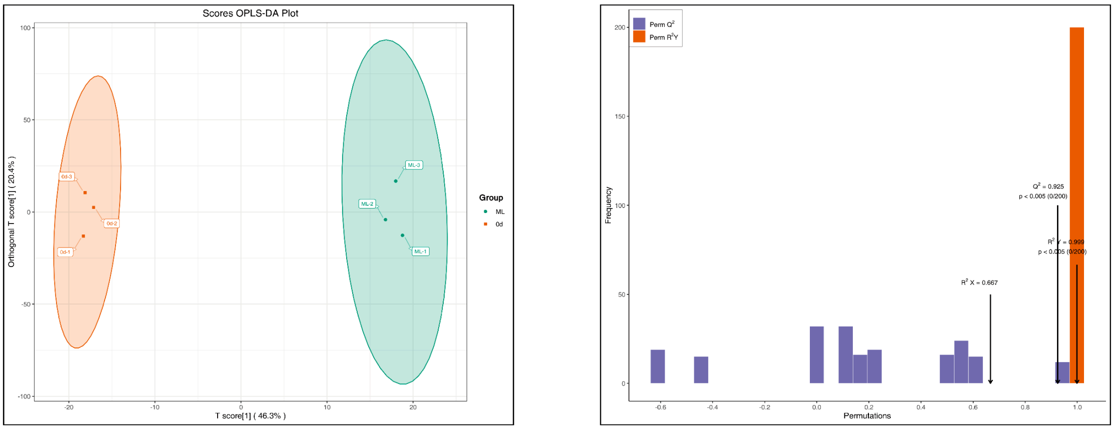

ML_vs_0d

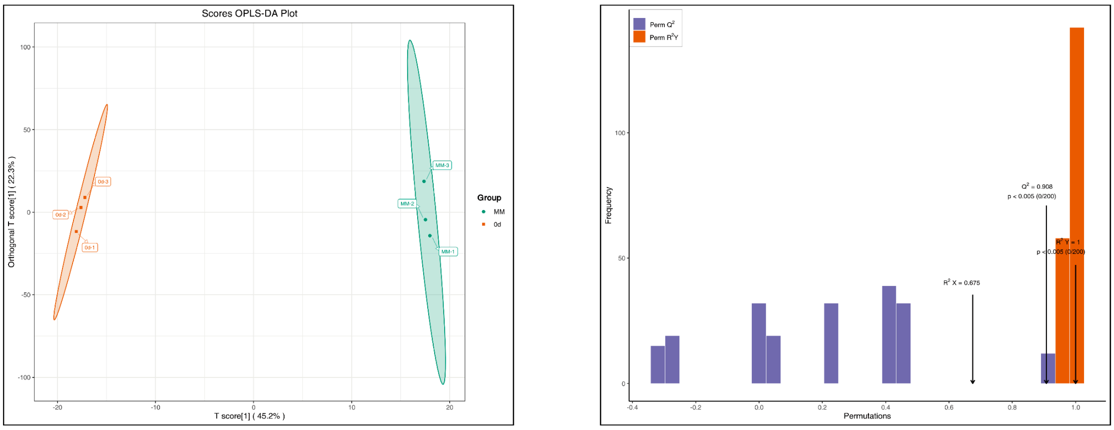

MM_vs_0d

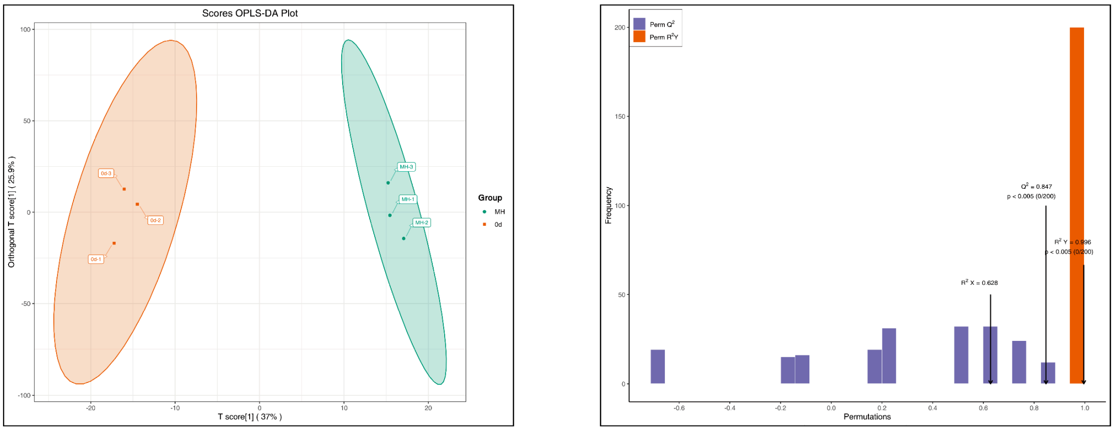

MH_vs_0d

Figure S2. OPLS-DA score plots of differential accumulation of VOCs (DAVs) between each treatment groups at days 360 and 0 and the corresponding permutation

test results for each model.

Table S1. Viscosity characteristics of millet at day 0.

PT (°C)	PV (cP)	TV (cP)	FV (cP)	BD (cP)	SB (cP)
80.12 ± 0.85	2197.17 ± 96.75	1248.70 ± 106.14	4872.65 ± 33.09	948.47 ± 9.39	2675.48 ± 63.66

Table S2. Two-way ANOVA of the effects of ST, GMC and their interaction on the oxidative indicators and quality components of millet under different storage periods.

**Parameter**	**Time (days)**	**ST**		**GMC**		**ST × GMC**	
		F-value	*p*-value	F-value	*p*-value	F-value	*p*-value
MDA	10	28.042	<0.001***	9.167	0.002**	0.899	0.485
	30	38.371	<0.001***	11.49	0.001**	0.891	0.489
	60	41.983	<0.001***	13.142	<0.001***	0.869	0.502
	150	43.891	<0.001***	13.496	<0.001***	0.787	0.548
	360	43.78	<0.001***	13.43	<0.001***	0.76	0.567
EC	10	3.167	0.066	0.667	0.526	0.917	0.476
	30	12.5	<0.001***	2	0.164	1.75	0.183
	60	17.429	<0.001***	5.429	0.014*	0.5	0.736
	150	18.086	<0.001***	5.743	0.012*	0.17	0.95
	360	26.41	<0.001***	8.04	0.003**	0.41	0.802
CAT	10	34.488	<0.001***	1.953	0.171	1.683	0.198
	30	20.926	<0.001***	1.346	0.285	0.059	0.993
	60	20.224	<0.001***	1.838	0.188	0.364	0.831
	150	34.128	<0.001***	2.173	0.143	0.899	0.485
	360	47.76	<0.001***	3.67	0.046*	1.89	0.156
YP	10	7.474	0.004**	3.114	0.069	0.514	0.726
	30	11.03	0.001**	3.622	0.048*	1.508	0.242
	60	7.459	0.004**	3.812	0.042*	1.547	0.231
	150	15.278	<0.001***	7.008	0.006**	2.359	0.092
	360	19.5	<0.001***	7.56	0.004**	2.38	0.091
CP	10	0.359	0.703	2.066	0.156	0.176	0.948
	30	10.366	0.001**	3.077	0.071	10.366	0.001**
	60	11.071	0.001**	6.964	0.006**	0.787	0.548
	150	11.621	0.001**	0.241	0.788	0.358	0.835
	360	19.44	<0.001***	1.92	0.175	0.16	0.954
SS	10	2.248	0.134	2.714	0.093	0.23	0.918
	30	1.449	0.261	1.853	0.186	0.36	0.834
	60	6.39	0.008**	5.167	0.017*	0.16	0.956
	150	13.732	<0.001***	4.119	0.034*	0.491	0.742
	360	42.48	<0.001***	4.1	0.034*	0.4	0.803

Abbreviations: MDA, malondialdehyde; EC, electrical conductivity; CAT, catalase; YP, yellow pigment; CP, crude protein; SS, soluble sugar. *, ** and *** indicate significance at $p < 0.05$, 0.01 and 0.001 levels, respectively.

Table S3. Two-way ANOVA for the effects of ST, GMC and their interaction on the viscosity characteristics of millet.

Parameter	ST		GMC		ST × GMC	
	F-value	*p*-value	F-value	*p*-value	F-value	*p*-value
PT	4.54	0.025*	0.02	0.981	2.80	0.057
PV	46.64	<0.001***	2.37	0.122	1.08	0.397
TV	28.70	<0.001***	0.11	0.896	1.60	0.217
FV	0.16	0.854	0.53	0.597	0.86	0.506
BD	9.68	0.001**	1.56	0.237	0.89	0.488
SB	30.91	<0.001***	4.14	0.033*	0.95	0.461

Abbreviations: PT, pasting temperature; PV, peak viscosity; TV, trough viscosity; FV, final viscosity; BD, breakdown value; SB, setback value. *, ** and *** indicate significance at $p <$ 0.05, 0.01 and 0.001 levels, respectively.

Table S4. Two-way ANOVA for the effects of ST, GMC and their interaction on major VOCs in millet.

	ST		GMC		ST × GMC	
	F-value	*p*-value	F-value	*p*-value	F-value	*p*-value
3-Octen-2-one	17.466	<0.001***	11.161	0.001**	11.193	<0.001***
Dodecanenitrile	2.694	0.095	6.540	0.007**	1.802	0.172
2,2,4-Trimethyl-1,3-pentanediol diisobutyrate	83.601	<0.001***	150.478	<0.001***	47.328	<0.001***
(Z)-4-Heptenal	29.258	<0.001***	10.471	0.001**	45.325	<0.001***
2-Methyl-6-(methylthio)-pyrazine	1.404	0.271	0.571	0.575	5.740	0.004 **
δ -Cadinene	12.561	<0.001***	14.772	<0.001***	2.168	0.114
(E)-4-Heptenal	29.258	<0.001***	10.471	0.001**	45.325	<0.001***
2-Methyl-3-(methylthio)-pyrazine	1.404	0.271	0.571	0.575	5.740	0.004 **

*, ** and *** indicate significance at $p < 0.05$, 0.01 and 0.001 levels, respectively.
